# Drawing the Line: From U-Net-Based Glioblastoma Segmentation to Machine Learning-Driven Survival Prediction

**DOI:** 10.3390/medsci14010119

**Published:** 2026-03-03

**Authors:** Costin Chirica, Bogdan-Ionuț Dobrovăț, Sabina-Ioana Chirica, Oriana-Maria Onicescu, Andreea Rotundu, Emilia-Adriana Marciuc, Laura-Elena Cucu, Daniela Pomohaci, Răzvan-Constantin Anghel, Mihaela-Roxana Popescu, Alexandra Maștaleru, Danisia Haba, Maria Magdalena Leon

**Affiliations:** 1 Faculty of Medicine, Grigore T. Popa University of Medicine and Pharmacy, 16 Universității Str., 700115 Iasi, Romania; 2 Faculty of Dental Medicine, Grigore T. Popa University of Medicine and Pharmacy, 16 Universității Str., 700115 Iasi, Romania; 3 Faculty of Computer Science, Alexandru Ioan Cuza University, 700506 Iasi, Romania

**Keywords:** glioblastoma, artificial intelligence, tumor segmentation, predictive modelling, survival prediction, machine learning

## Abstract

**Background/Objectives**: Glioblastoma (GB) remains the most prevalent primary malignant brain tumor in adults, characterized by its aggressive nature and poor prognosis. The present study endeavored to contribute to the development of advanced computational tools for neuro-oncology by integrating artificial intelligence (AI)-based segmentation and multi-model machine learning (ML) approaches. **Methods**: A retrospective analysis was conducted on patients with GB. AI-driven algorithms were utilized to perform volumetric segmentation of GB. These quantitative metrics were subsequently integrated into a multi-model ML framework to analyze correlations with patient survival and evaluate the predictive accuracy of the resulting models. **Results**: A total of 79 patients were ultimately included in the study after meeting all eligibility criteria. The results showed that larger GB tumors were associated with shorter post-treatment survival. Necrotic patterns within GB tumors impacted patient survival rates and response to therapy. Quantitative volumetric analysis of tumor enhancement, shape features, and morphological metrics were associated with patient outcomes. The Neural Network remained the top ML model performer overall for discrimination, but the Random Forest model also showed strong practical performance. **Conclusions**: As a summary, our study contributes to the development of advanced computational tools for neuro-oncology by integrating AI-based segmentation and multi-model ML approaches, and the results highlight the importance of imaging biomarkers in understanding GB prognosis.

## 1. Introduction

Brain tumors encompass a wide range of abnormal intracranial cellular growth patterns and include some of the most aggressive types of cancer. Glioblastoma (GB) remains the most common primary brain tumor in adults, characterized by its malignant and fatal nature, with an incidence of 2–3 cases per 100,000 people [1,2,3].

The latest Classification of Tumors of the Central Nervous System (CNS) published by the World Health Organization (WHO) in 2021 establishes a histopathologically distinct diagnosis for GB. Currently, GB is identified as a diffuse astrocytic tumor in adults that is IDH-wildtype [4,5,6].

Evidence suggests that GB is predominantly prevalent in the elderly population, with a median diagnostic age of 64 years. Moreover, it is most common in male patients and it is characterized by increased cell division, invasiveness, neoangiogenesis, and central necrosis [7,8,9]. Consequently, GB has an extremely poor prognosis, being an clinical entity with a median survival time of 15–16 months and a 5-year survival rate of approximately 5–10% [10]. One of the primary factors contributing to the incurability of GB is its high rate of recurrence, which is likely due to the unfeasibility of a complete surgical removal of the tumor mass [11].

Nowadays, recent reports have analyzed comprehensive genomic and epigenomic characteristics to understand long-term survival in patients with GB [12,13]. Long-term survivors (LTS) of GB are defined as those who live longer than 2 years post-diagnosis [14]. It is generally accepted that there is a subset of GB patients who live longer than 2 years and are classified as LTS, with 5% to 13% surviving an exceptional 5 years [15]. The rarity of LTS is indicated by the sharp decline in survival rates over time, with only about 6% of patients surviving up to 5 years [16]. Thus, despite the aggressive nature of GB, there is a subset of patients who achieve long-term survival.

Despite significant advancements in defining molecular subgroups, the etiology of GB remains unknown. Less than 20% of patients exhibit a familial predisposition to cancer. Specific risk factors, such as being exposed to radiation, having a genetic predisposition, and having specific inherited disorders, may contribute to the development of the disease [17].

A hallmark of its biological aggression is that GB develops by infiltrating the apparently normal tissue surrounding the tumor, namely the peritumoral brain zone [11,18]. The ability of GB to invade surrounding brain tissue and expand quickly are its most distinctive characteristics [19]. However, reflecting the underlying mass effect and increased intracranial pressure, symptoms frequently include headaches, convulsions, altered cognition, and neurological impairments [20]. The majority of the patients with GB are diagnosed by contrast-enhanced magnetic resonance imaging (MRI) and subsequently confirmed by histological diagnosis, but less than 60% of patients over the age of 70 receive the pathological confirmation [21].

According to current neuro-oncological guidelines, the standard treatment for this tumor type is maximal safe resection, followed by radiotherapy and concomitant chemotherapy with temozolomide, as well as adjuvant treatment consisting of 6 cycles of temozolomide. Among the patients expected not to tolerate standard radiotherapy, hypofractionated radiotherapy may be used [22]. However, the current conventional therapeutic modalities offer only a limited amelioration in the clinical outcomes and unfortunately recurrence is predicted in 90% of patients within 12 months following the diagnosis [23,24,25].

Therefore, in clinical practice, early evaluation of GB requires neuroimaging analysis obtained through contrast-enhanced MRI. The classic MRI finding of GB is an irregularly shaped, rim-enhancing lesion surrounding a central hypointense necrotic core. Involvement of the deep white matter and the corpus callosum is common. Depending on the tumor volume and perilesional edema, the intra-axial expansive process can displace midline structures and present a significant mass effect on normal brain matter [17,26].

In the modern era of neuroimaging, studies in the field place emphasis on the importance of an early and accurate GB imaging diagnosis, as well as on the detailed analysis of imaging pattern, in order to improve the prognosis paradigm and permit an efficient patient management [27]. Therefore, GB imaging holds the ability to contribute with precise and significant data for a highly needed progress in the treatment alternatives for these patients as well as for the monitoring of the tumor progression [28].

However, craniocerebral contrast-enhanced MRI remains the pivotal paraclinical investigation in GB, providing essential parameters which can be investigated in relation to overall survival (OS). These include the tumor volume itself, the volume of the contrast-enhancing and non-enhancing tumor, the volume of the perilesional edema, the fractal parameters of necrosis, and geometric measures of the tumoral rim. Consequently, the volumetric parameters play a crucial role in predicting outcomes for GB patients, aiding in the identification of high-risk patients requiring treatment adjustments and further seeking alternative therapies for better results [29,30].

For this reason, pre-treatment data obtained via MRI has the potential to offer significant insights into understanding the tumor kinetics [31]. Moreover, artificial intelligence (AI) is a dynamically advancing field, with exciting results based on MRI images [32]. Numerous AI models have been the subject of current research and show the possibility of being integrated into clinical practice [33]. Thus, the application of deep learning (DL) and machine learning (ML) algorithms for MRI-based tumor segmentation and grading has yielded state-of-the-art results. These models exhibit exceptional performance, serving as critical tools for enhancing surgical strategies and augmenting therapeutic response [34].

Within this framework, our study was designed to quantify the potential correlations between the specific volumetric components of GB—including the solid tumor volume, the volume of the tumor necrosis, and the volume of the perilesional edema—as well as the OS rates in patients diagnosed with this highly aggressive cerebral pathology.

This approach seeks to evaluate the extent to which the tumor’s internal architecture and its micro- or macro-environmental impact—assessed via AI-imaging techniques—can serve as reliable predictive or prognostic markers.

Also, the goal of this research is to employ a multi-model approach, training multiple ML models to predict survival outcomes in patients with GB.

## 2. Materials and Methods

### 2.1. Study Overview

A retrospective cohort study was performed involving patients diagnosed with GB who underwent clinical management at the ‘Prof. Dr. N. Oblu’ Emergency Clinical Hospital (Iași, Romania) between 2019 and 2023.

The primary objective was to correlate tumor volumes, derived from pre-treatment MRI segmentation, with OS outcomes. Furthermore, the research aimed to integrate these volumetric and clinical datasets into predictive ML models. By leveraging these computational approaches, the study sought to develop robust prognostic tools designed to enhance clinical decision-making.

#### 2.1.1. Specific Objectives of the Study

To achieve these overarching goals, the study is structured around several interconnected specific objectives, beginning with the identification and retrospective analysis of a patient cohort diagnosed with GB and treated at the ‘Prof. Dr. N. Oblu’ Emergency Clinical Hospital, based on stringent clinical and histopathological inclusion criteria (which are detailed below).

Central to this process is the advanced imaging segmentation and volumetric quantification of pre-operative MRI scans, which enables the measurement of tumor sub-regions, specifically the necrotic core, enhancing tumor, and peritumoral edema. Subsequently, the research seeks to perform a clinical-radiological correlation assessment to determine the statistical relationship between these derived volumes and OS outcomes, thereby establishing their individual prognostic significance.

These quantitative datasets, alongside essential clinical variables such as age, sex, and tumor location (corpus callosum, frontal, parietal, temporal, and occipital), are then integrated into the development and training of ML models designed to generate robust survival predictions.

Finally, the study concludes with the validation and evaluation of these predictive models using high-fidelity metrics, including MSE, AUC and F1 score, to ensure their reliability in forecasting clinical progression and their potential utility in personalizing therapeutic strategies in neuro-oncology.

#### 2.1.2. Inclusion and Exclusion Criteria

The study cohort comprised adult patients (aged over 18 years at the time of diagnosis) with a histologically confirmed diagnosis of GB. Eligibility was restricted to individuals who underwent standard-of-care management, defined as maximal safe resection followed by adjuvant radiotherapy and chemotherapy. Furthermore, inclusion was predicated on the availability of high-quality, pre-treatment MRI scans suitable for precise volumetric analysis. Finally, participants were required to have comprehensive clinical records, specifically documented OS data with verified dates of diagnosis, mortality, or the last known follow-up.

Conversely, patients were excluded from the analysis if they had received any form of anti-tumor therapy prior to the baseline MRI acquisition or if the imaging quality was insufficient to allow for reliable volumetric assessment. The study also omitted individuals lost to follow-up or those for whom survival outcomes could not be accurately quantified. Additionally, the presence of other concurrent active primary malignancies served as a basis for exclusion. To ensure the integrity of the oncological data, the analysis also excluded patients who succumbed to non-oncological complications within 30 days of the surgical intervention.

### 2.2. Data Collection, Data Cleaning and Preprocessing

Clinical and radiological data were systematically compiled into a dedicated database. Collected variables included demographics (age at diagnosis and sex) and temporal data (dates of diagnosis and death). Radiographic parameters encompassed the topographic localization, the manual volumetric measurements, and the automated outputs from the AI model, including the total tumor volume and the sub-compartmental volumes (contrast-enhancing component, peritumoral edema, and necrosis).

All the collected parameters were systematically organized and stored in a digital database for subsequent statistical analysis. Columns were standardized and renamed for consistency prior to analysis.

The dates of the histopathological diagnosis and the times of death were used to determine the expected survival time and the date 1 April 2025 was used as a placeholder for missing death dates. The placeholder date did not influence the survival analysis calculations, as censored observations were properly flagged and their true follow-up duration was used. Duplicate patient identifiers were removed to ensure patient-level analyses. All analyses were performed on the cleaned dataset, by specific exclusion reasons (missing or inconsistent dates, incomplete imaging measures).

The primary outcome for survival analyses was OS (months) measured from histopathologic diagnosis to death. Survival durations are expressed in months and an event indicator was set to 1 for observed deaths and 0 for censored observations.

We derived multiple imaging biomarkers from the raw variables. Manual tumor volumes were normalized using the scalar π/6 to convert raw measurements into a standardized volume estimate. AI-derived measurements included the total estimated volume and the compartmental volumes (contrast, necrosis, edema). Ratios and differences were also computed. To reduce extreme influences, ratio variables were truncated at predefined caps.

### 2.3. MRI Acquisition Parameters and Protocols

The MRI examinations were performed using a General Electric Medical Systems (Waukesha, WI, USA)—Signa Explorer 1.5 Tesla scanner. The imaging protocol prioritized high-resolution 3D acquisitions, specifically involving sagittal 3D T2 FLAIR and post-contrast 3D T1W sequences (both FSPGR and non-FSPGR variants). Detailed acquisition parameters, including repetition time (TR), echo time (TE), and spatial resolution, are summarized in Table 1.

### 2.4. Manual Volumetric Assessment of the Tumor

The tumor volume was quantified using the ellipsoidal method, based on the principle that the morphology of brain pathology is most accurately approximated by an elliptical geometry rather than a cubic one [35].

The protocol involved a two-stage process. First, the 3D T1 weighted contrast-enhanced (CE-T1W) acquisitions underwent rigorous spatial normalization. This entailed aligning the medio-sagittal line in the coronal and axial planes and the bi-commissural axis in the sagittal plane to ensure anatomical consistency. Secondly, the maximum orthogonal diameters were measured across all three planes. The product of these measurements was subsequently adjusted by the ellipsoidal constant of 0.52 to derive the final volumetric data [36].

In order to guarantee the robustness of the findings and minimize subjective bias, all measurements were performed independently by two senior neuroradiologists.

### 2.5. Automated Volumetric Segmentation of the Tumor

Automated volumetric analysis was performed using the mediaire software solutions mdbrain 2.7 (distributed in Romania via Supermedical), a class II CE-certified medical device. The software employs a DL algorithm based on a 3D convolutional neural network with a U-Net architecture [36,37].

To facilitate a comprehensive segmentation, CE-T1W and T2-weighted Fluid-Attenuated Inversion Recovery (T2W FLAIR) sequences were co-registered and integrated.

This multi-sequence approach allows for the simultaneous characterization of the solid enhancing component and the central necrotic core (identifiable via CE-T1W), alongside the peripheral vasogenic edema (characterized by hyperintense signal on FLAIR without contrast enhancement).

**Figure 1:**
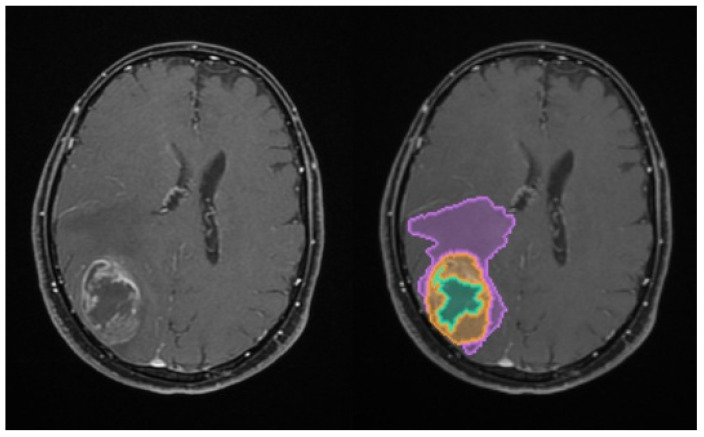
Illustration of the automated volumetric analysis of contrast-enhanced T1-weighted axial MRI sequences using the mdbrain software (scale 1:20). The segmented regions include the necrotic core (green), the contrast-enhancing tumor component (orange), and the perilesional edema (purple) (approval was obtained from the Ethics Committee of the University of Medicine and Pharmacy “Grigore T. Popa” Iasi).

The total tumoral volume was thus partitioned into these three distinct sub-compartments, with the resulting Digital Imaging and Communication in Medicine (DICOM)-processed outputs illustrated in Figure 1.

### 2.6. Statistical Analysis

The distributions of continuous variables were inspected using histograms and kernel density estimates. Pairwise scatterplots and correlation assessments were used to identify strong associations between imaging-derived features (for example, AI edema vs. AI necrosis vs. volume measures) and with survival. Summary statistics (mean, median, standard deviation, minimum, maximum and counts) were computed overall and stratified by key categorical variables (tumor side, precise localization, gender).

We compared continuous outcomes between groups using parametric or nonparametric tests depending on the number of groups and distributional properties. For two-group comparisons we applied the Student’s *t*-test or Mann–Whitney U test; for series of more than two groups we used one-way ANOVA or Kruskal–Wallis tests. The value of *p* ≤ 0.05 was considered statistically significant.

### 2.7. Survival Analysis Methods

The Kaplan–Meier estimator was used to describe survival functions for the overall cohort and for the stratified groups [38]. Survival curves and median survivals with 95% confidence intervals were computed and plotted. Log-rank tests were used for unadjusted comparisons where appropriate [39]. To assess the proportional hazards assumption graphically, complementary log–log (cloglog) plots of log(−log(S(t))) versus log(time) were generated for major strata and visually inspected.

Multivariable Cox proportional hazards models were fit to estimate hazard ratios (HR) associated with demographic and imaging-derived covariates [40]. Categorical predictors were dummy-encoded (drop-first), and models were fit only when the sample size for the covariate grouping exceeded a minimum threshold to avoid unstable estimates. Duration and event variables were constructed as described above; hazard ratios with 95% confidence intervals and *p*-values are reported.

### 2.8. Predictive Modelling

The integration of AI into medical research has revolutionized the field of oncology. In the context of GB, by leveraging high-dimensional imaging data and clinical information, ML algorithms can extract valuable insights into patient prognosis. In this study, we harnessed the power of AI to explore the potential correlations between tumor volumetry and survival outcomes in GB patients, laying the groundwork for the development of more accurate predictive models that can inform clinical decision-making.

#### 2.8.1. Regression–Survival in Months

We modeled the problem as a regression estimator. For handling the large number of features compared to the total patients in the study, we chose random forest (RF) models.

For creating the RF model, the dataset is split into sub-datasets. Each sub-dataset is used to construct a decision tree (DT), in the end forming a forest of many trees. The strength of RF lies in its assembly of multiple DTs, enabling it to achieve higher and more robust performance by better generalizing across the dataset.

To predict continuous survival (months) we compared four model families: RF Regressor (*n*_estimators = 100, max_depth = 2; class_weight = ‘balanced’), XGBoost Regressor (*n*_estimators = 100, max_depth = 1; scale_pos_weight = class_ratio), k-Nearest Neighbors (KNN) Regressor (*n*_neighbors = 3, weights = ‘distance’), and a fully connected Neural Network (sequential model with a hidden layer of 8 neurons) implemented in Keras [41,42,43,44].

The feature set used for model development is: gender, age at diagnosis, manual computed volume normalised, AI model estimated volume, AI model contrast volume, AI model necrosis volume, AI model edema volume, all volume ratios, tumor location, and tumor side, which are the result of feature selection from correlation with the survival features, or are proven by literature [34].

A cross-validation holdout was used with a 20% testing and 80% training split. Model performance was evaluated on the held-out test set using mean absolute error (MAE), mean squared error (MSE) and $R^{2}$.

#### 2.8.2. Classification–6-Month Threshold

For binary prediction of survival ≥ 6 months we compared Random Forest Classifier (*n*_estimators = 100, max_depth = 2; class_weight = ‘balanced’), XGBoost Classifier (*n*_estimators = 100, max_depth = 1; scale_pos_weight = class_ratio), k-Nearest Neighbors Classifier (*n*_neighbors = 3, weights = ‘distance’), and a Neural Network (sequential model with a hidden layer of 8 neurons) trained with class weights. The same train/test split strategy described above was used. The class ratio split was used in training to adapt to the imbalance. Classification performance metrics included accuracy, precision, recall, F1 score and area under the ROC curve (AUC). Confusion matrices were reported to illustrate the trade-off between sensitivity and specificity.

### 2.9. Model Explainability

Model-agnostic local explanations were produced using LIME (local interpretable model-agnostic explanations) for tabular data. An explainer was initialized on the training set and explanations were generated for a sample of test instances; feature contributions were aggregated across instances to produce mean absolute importances per model.

### 2.10. Software, Packages and Reproducibility

Analyses were conducted in Python 3.13 using pandas, NumPy, SciPy, scikit-learn, xgboost, Keras/TensorFlow, lifelines, seaborn, matplotlib, lime and TableOne. Random seeds were set where models support deterministic seeds.

## 3. Results

From an initial screening of 695 patients diagnosed with GB during the 2019–2023 period, several cases were excluded based on stringent imaging requirements. A significant limiting factor was the lack of access to raw DICOM data for patients whose initial MRI scans were performed at external institutions. Moreover, cases exhibiting significant motion or susceptibility artifacts were omitted, as such technical limitations hindered the accuracy of automated AI volumetric processing.

A total of 79 patients were ultimately included in the study after meeting all the eligibility criteria. The baseline characteristics of the study cohort are summarized in Table 2.

### 3.1. Group Comparisons

**Figure 2:**
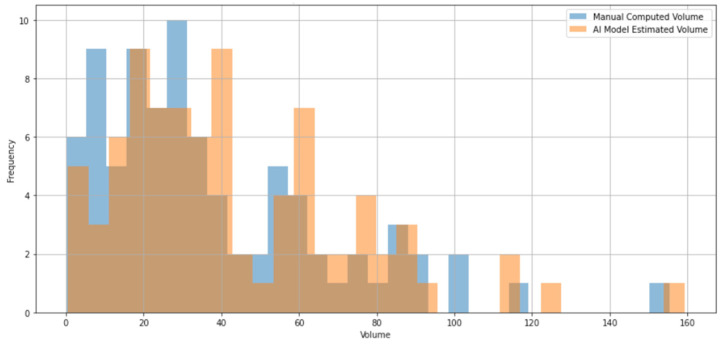
Comparison of volume distributions: manual computed tumoral volume vs. AI model estimated tumoral volume.

Histograms of manual normalized tumor volume and AI-estimated volume are shown in Figure 2, demonstrating right-skewness and overlapping.

**Figure 3:**
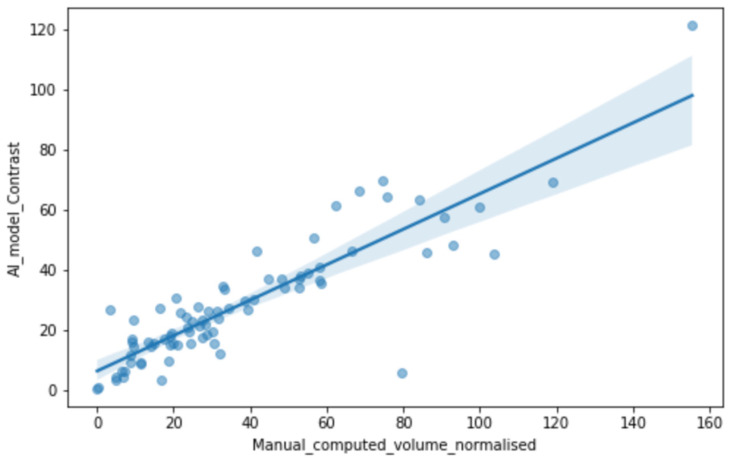
Scatter plot of manual computed volume normalized vs. AI model contrast tumoral volume estimated and their correlation.

Pairwise scatterplots (Figure 3) showed a strong positive correlation between the AI-estimated volume and the manual normalized volume (Spearman ρ = 0.93, *p* value = 2.57 × 10^−38^) and low correlation between the AI edema and the AI necrosis (*p* value = 0.26). These relationships motivated inclusion of both volume and compartment ratios in downstream models.

The comparison of edema values derived from the AI based model according to tumor location (left/central/right) demonstrated a statistically significant difference among groups (one-way ANOVA, *p* = 0.049), with centrally located tumors showing lower AI-derived edema values compared with laterally located tumors.

### 3.2. Survival Analysis

**Figure 4:**
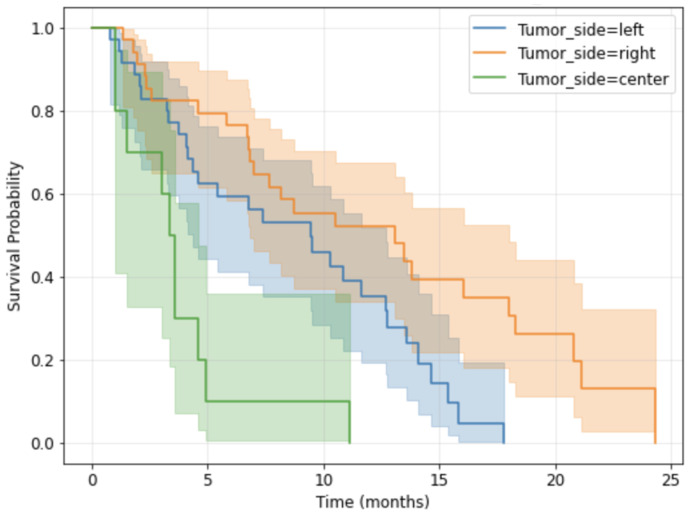
Kaplan-Meier Survival curves by tumor side.

The survival outcomes were stratified by tumor laterality (left, center, right), as detailed in Table 3. The statistical tests (Cox Regression Results, left vs. center HR = 0.32822, *p* value = 0.00369, right vs. center HR = 0.16140, *p* value = 0.00001) indicated a significant difference in median survival between tumor sides, central tumors being the most aggressive (Figure 4).

**Figure 5:**
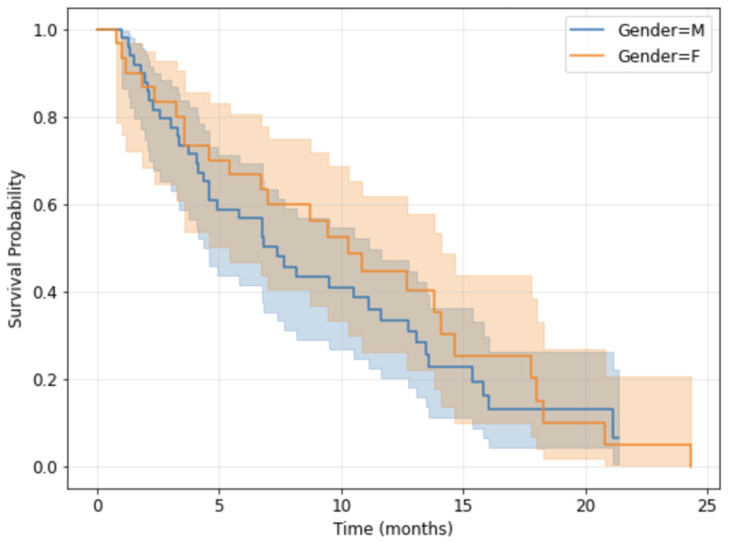
Kaplan-Meier survival curves by gender.

**Figure 6:**
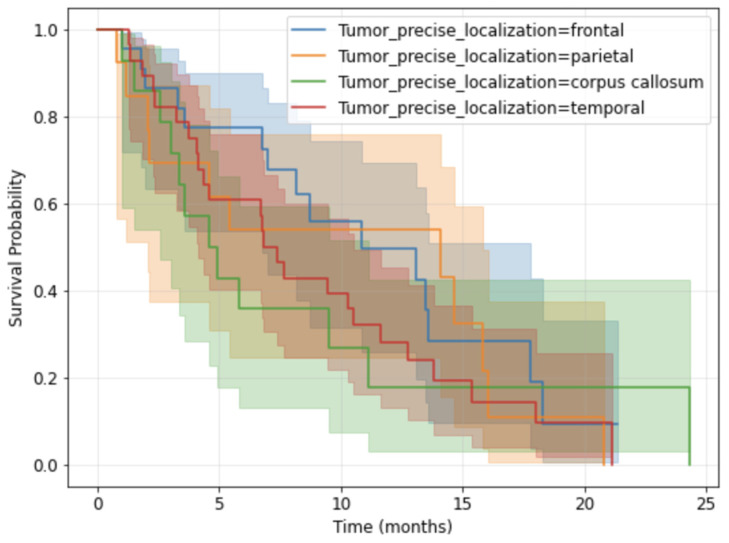
Kaplan-Meier survival curves by tumor precise localization.

The Kaplan–Meier survival curves for the overall study population stratified by sex are presented in Figure 5, while the ones stratified according to the precise tumor localization are shown in Figure 6. The median overall survival for the cohort was 8.16 months (95%, confidence interval 2.3–14.63).

A multivariable Cox proportional hazards analysis was performed, adjusting for age at diagnosis, manual normalized volume, and AI-estimated volumes. Significant independent predictors associated with decreased survival rates included higher manual normalized volume (HR = 1.01057, *p* = 0.01906), higher AI model contrast volume (HR = 1.01811, *p* = 0.01106), and higher AI model estimated volume (HR = 1.01052, *p* = 0.03193).

### 3.3. Regression Models Predicting Continuous Survival Performance

The four trained regression models, as described in the Section 2, were used to predict continuous survival outcomes expressed in months. Performance on the held-out test set is summarized in Table 4. The XGBoost regressor produced MAE (mean absolute error) = 5.315 months, MSE (mean squared error) = 44.779; The Random Forest and KNN models demonstrated slightly inferior predictive performance, whereas the Neural Network exhibited evidence of overfitting, as reflected by suboptimal MSE performance.

The classification model, a simplified approach to the regression model, is less sensitive to large errors in exact month estimates, which degrades regression performance. In practice, this simplification improved predictive performance and achieved higher discrimination (AUC) than the regression models’ continuous-error metrics. We therefore present the classification results alongside regression; however, this approach misses information regarding finer-grained survival differences, which require a prespecified threshold (6 months here) chosen for its clinical relevance.

### 3.4. Classification Models (6-Months Survival) Performance

**Figure 7:**
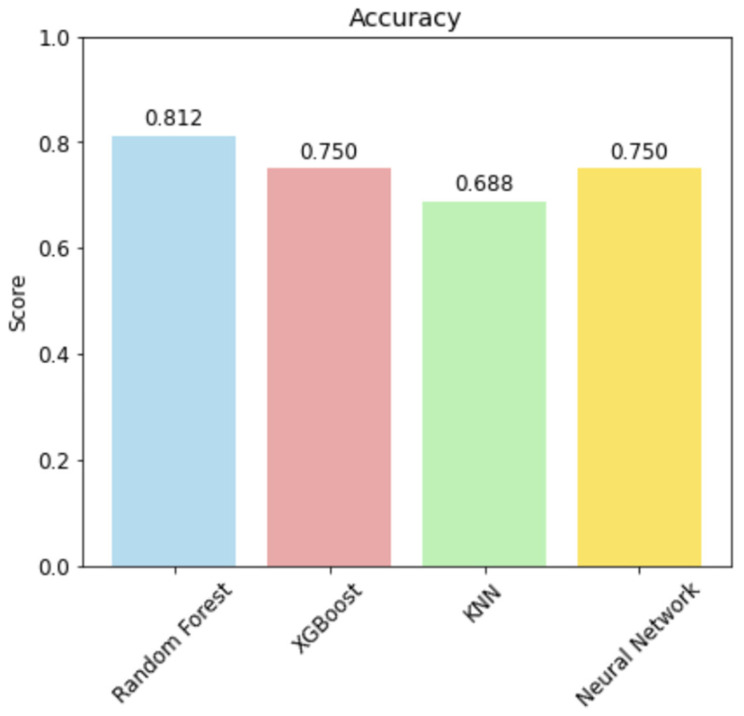
Accuracy of the classification models (hyperparameters were chosen using grid search).

**Figure 8:**
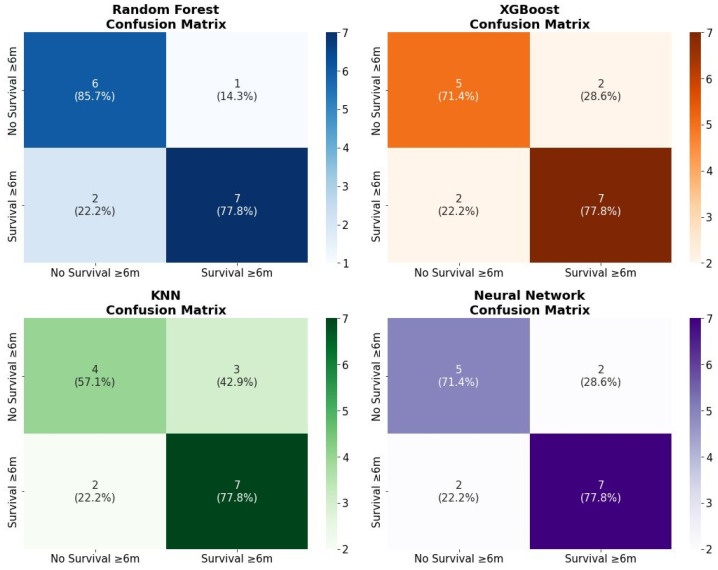
Confusion Matrix for the classification models.

For binary prediction of survival ≥ 6 months we compared Random Forest Classifier (Cross-Validation, 95% CI: 0.554–0.889), XGBoost Classifier (Cross-Validation, 95% CI: 0.676–0.839), k-Nearest Neighbors Classifier (Cross-Validation, 95% CI: 0.575–0.768), and a Neural Network (Cross-Validation, 95% CI: 0.433–0.808) trained with class weights. Model hyperparameters were chosen using grid search (e.g., k = 3 for KNN). Classification performance metrics included accuracy, precision, recall, F1 score and area under the ROC curve (AUC). Confusion matrices with absolute counts and row percentages were reported to illustrate the trade-off between sensitivity and specificity (see Figure 7 and Figure 8).

### 3.5. Feature Importance and Explainability

Feature importances indicate that the top predictors included AI-model necrosis, manually normalized volume, and AI edema volume. Aggregated LIME explanations corroborated the importance of these features across multiple model families. The most relevant features for each model are also consistent with our intuition, as shown in Table 5.

## 4. Discussion

GB constitutes the most prevalent primary brain malignancy in adults, distinguished by hallmark features such as extensive necrosis and diffuse infiltration into perilesional parenchyma. These pathological attributes validate its aggressive clinical profile, marked by pronounced resistance to standard-of-care therapies and a high recurrence rate. Consequently, the prognosis remains dismal, underscoring the imperative for expedited diagnosis and tailored therapeutic interventions to optimize patient management [29,45,46].

Nowadays, progress is being made in identifying prognostic factors derived from MRI features of GB, promising a targeted, personalized approach to achieve positive long-term outcomes for GB patients. In this regard, Sanghani and colleagues [47] evaluated 13 tumor shape features for predicting OS in GB patients. Significant 3D shape features, including the bounding ellipsoid volume ratio, sphericity, and spherical disproportion, derived from both FLAIR and contrast-enhanced T1-weighted masks, were prognostic indicators of OS in GB patients. These features were more effective when derived from the contrast-enhanced T1-weighted sequence compared to the FLAIR sequence. Similar to our study, their research used univariate and multivariate Cox regression analyses, along with Kaplan-Meier survival curves, in order to assess the prognostic value of these shape features for OS in GB patients. Their findings suggest that irregular tumor shapes are associated with poor survival, whereas regular tumor shapes are associated with prolonged survival.

However, the quantification of tumor volume as a prognostic marker for GB was reported by Reeves and Marks in 1979 [48]. Their research indicated that larger GB are associated with shorter post-treatment survival. Even today, the volume of GB measured by MRI is considered one of the most significant prognostic factors in consequence of its strong correlation with disease progression and patient survival. Our findings reveal that larger tumors are usually associated with a poorer prognosis, as they indicate a higher tumor burden and a greater risk of impairing essential brain functions [49,50].

Regarding tumor volume, a recent study [51] focuses on the preoperative MRI evaluation and provides insights into potential prognostic indicators and the importance of accurate GB volume measurements for predicting patient outcomes. The cohort consisted of 44 patients, predominantly male, with a median age of 58.15 years. Palpan Flores et al. used various volumetric methods in order to measure tumor size and compartments, showing that GB volumes were optimal neuroimaging biomarkers for predicting survival in GB patients. It is remarkable that OS was associated with age ≥ 65 years and a tumor volume measured in FLAIR-T2 of ≥2000 mm^2^ or ≥60 cm^3^.

Moreover, understanding the dynamics of contrast-enhancing tumor volume over time can provide essential awareness of the aggressiveness of the disease and guide personalized treatment strategies for patients with GB.

In this regard, Auer and colleagues [52] proposed turning the spotlight on quantitative volumetric analysis, recognizing the limitations of the existing methods and the need for more robust prognostic tools. Their research included a cohort of 49 patients diagnosed with GB, with the objective of examining the possibility of 3D volumetric measurements of tumor enhancement as a predictive radiomic imaging biomarker. Results indicate a substantial correlation between high contrast-enhanced tumor volume and shortened survival, providing a baseline for considering 3D volumetric approaches as predictive biomarkers in GB. Thus, this study provides a glimpse into the prospective resolution of the ongoing discussion on the effectiveness of different measurement approaches, illustrating the potential of 3D volumetric quantification as a clinically relevant tool for GB prognosis.

The GB volume at diagnosis provides prognostically relevant information and supports clinicians in making appropriate, individualized management decisions. In addition to tumor volume, the presence and extent of necrosis—an imaging hallmark of GB—also influence prognosis and therapeutic planning and therefore warrant particular attention.

Necrosis is a diagnostic hallmark of GB, as its presence is identified in over 85% of the cases [53]. Researchers have long been intrigued by the relationship between necrosis and GB progression because understanding this connection could lead to advances in treatment options for patients. Studies have shown that necrosis within GB tumors can impact patient survival rates as well as their response to therapy, highlighting the importance of further investigating this phenomenon [54,55].

Unquestionably, necrotic patterns in GB that are linked to patient survival have been the focus of extensive research in recent years. Understanding the relationship between necrotic areas within GB tumors and patient outcomes is vitally important for developing more appropriate treatment options. In this regard, Liu and colleagues [56] investigated the prognostic role of necrotic patterns in GB using fractal dimension (FD) and lacunarity analyses derived from MRI data. The authors analyzed clinical and MRI data of 95 GB patients and calculated the FD and lacunarity of the necrosis by fractal analysis. The study revealed significant correlations between lower FD values and larger lacunarity values and a decline in PFS (*p* = 0.006 and *p* = 0.012, respectively) and OS (*p* = 0.008 and *p* = 0.005, respectively) in patients with GB. This demonstrates that the fractal parameters of necrosis in GB can predict patient survival and are accordingly linked to the biological processes concerning tumor necrosis. In addition, lacunarity was found to be associated with the suppression of apoptosis—and necrosis-related biological processes, shedding light on the importance of necrosis in GB prognosis and the molecular pathways that lead to it.

As a further matter, Curtin and collaborators [57] explored the use of morphological metrics, specifically lacunarity and FD, for the analysis of GB abnormalities on MRI and their connection to patient survival. The study found significant correlations between these morphological metrics and patient outcomes, with the shape of T2/FLAIR (fluid-attenuated inversion recovery) abnormalities showing the strongest link to OS.

Most recently, a study published in 2024 by Ma et al. [58], involving a cohort of 150 patients, demonstrated that imaging-defined necrosis was strongly associated with glioblastoma and with specific genetic alterations, including 1p/19q non-codeletion and homozygous deletion of CDKN2A/B, and functioned as an independent prognostic marker for tumor outcome. This research emphasizes the critical role of necrosis, particularly imaging necrosis, in evaluating GB, providing valuable insights for an accurate diagnosis and prognosis. Additionally, in this study, dynamic contrast-enhanced-MRI metrics demonstrated a high diagnostic efficiency in identifying tumor necrosis.

However, some studies discuss the anatomical features of GB on imaging, including cysts, necrotic changes, and the extent of edema, and emphasize the importance of complete resection for better outcomes. Other studies focus on the prognostic value of morphological MRI-based imaging biomarkers, such as tumor volume and surface regularity, for predicting survival in GB patients. The common direction is to highlight the significance of specific imaging characteristics in understanding and predicting outcomes for patients with GB. For example, in recent years, numerous studies have focused on the link between perilesional edema and the prognosis in GB patients (Table 6).

Overall, by incorporating tumor volumes into ML models for prognostic evaluation, our study revealed that the Neural Network achieved superior discriminative performance, while the Random Forest displayed robust clinical relevance in confusion matrix assessments. In particular, the Random Forest achieved a favorable balance between true-positive and true-negative classifications in the held-out dataset, suggesting significant utility in clinical contexts where specific error profiles are critical, such as minimizing the risk of misclassifying high-risk patients.

From a practical perspective, the Neural Network may be considered the primary model for survival ranking and prediction, while the Random Forest represents a complementary alternative when greater interpretability of decision thresholds or increased robustness to noise in tabular features is desired.

The findings of our study carry significant clinical implications for the diagnosis and management of patients with GB. Nevertheless, certain limitations warrant consideration. The retrospective nature of the analysis, coupled with a limited cohort size, may affect the robustness of the conclusions. Furthermore, the single-center design potentially limits the generalizability of the results. Consequently, these findings should be interpreted as an exploratory analysis of imaging-biomarker associations rather than a definitive clinical prediction model. A rigorous multivariate survival analysis necessitates the integration of critical molecular and clinical variables—specifically methylguanine-DNA methyltransferase (MGMT) methylation status and Karnofsky Performance Status—to effectively account for confounding factors. Therefore, further validation through prospective, multicenter research is required to confirm these results and facilitate their implementation into routine clinical practice.

## 5. Conclusions

As a summary, our study contributes to the development of advanced computational tools for neuro-oncology by integrating AI-based segmentation and multi-model ML approaches, and the results highlight the importance of imaging biomarkers in understanding the prognosis of GB.

Future work may reasonably explore model combination strategies, such as ensemble or stacked approaches, or selection of the optimal model based on predefined clinical priorities (e.g., optimization of sensitivity versus specificity), prior to external validation.

## Figures and Tables

**Table 1:** MRI data acquisition.

Sequence	TR	TE	Slice Thickness	Spacing
3D T2 FLAIR	6502–7002	118.597–122.618	1.8 mm	0.9 mm
post-contrast 3D FSPGR T1W/3D T1W	19.892/9.464	4.2	1.8 mm	0.9 mm

3D FSPGR T1W: Three-Dimensional Fast Spoiled Gradient Recalled Echo T1-Weighted; 3D T2 FLAIR: Three-Dimensional T2 Fluid-Attenuated Inversion Recovery; TE: Echo Time; TR: Repetition Time.

**Table 2:** The characteristics of the patients included in the study.

		Overall
N	N/A	79
Age at diagnosis, mean (SD)	N/A	59.9 (11.6)
Survival months, mean (SD)	N/A	8.6 (6.0)
Gender, N (%)	F	30 (38.0)
	M	49 (62.0)
Tumor precise localization, N (%)	corpus callosum	14 (17.7)
	frontal	22 (27.9)
	occipital	2 (2.5)
	parietal	13 (16.5)
	temporal	28 (35.4)
Tumor side, N (%)	center	10 (12.7)
	left	35 (44.3)
	right	34 (43.0)
Manual computed volume normalised, median [Q1, Q3]	N/A	28.4 [16.6, 53.0]
AI model estimated volumes, median [Q1, Q3]	total	34.6 [20.1, 60.4]
	contrast	23.5 [15.4, 36.7]
	necrosis	10.8 [4.0, 20.0]
	edema	76.0 [48.6, 118.9]

SD: standard deviation; N: number; N/A: not applicable.

**Table 3:** Overall survival for each tumor side.

	OS (In Months)					
Tumor Side	Mean	Median	SD	Min	Max	Count
center	3.766667	3.466667	2.930428	1.000000	11.133333	10
left	7.900000	7.366667	5.010329	0.766667	17.800000	35
right	10.795098	9.616667	6.607063	1.333333	24.300000	34

Max: maximum value; Min: minimum value; OS: overall survival; SD: standard deviation.

**Table 4:** Performance of machine learning models.

	MAE	MSE
Random Forest	5.038 (95%, CI: 4.078–5.997)	36.292 (95%, CI: 30.394–42.190)
XGBoost	5.315 (95%, CI: 4.412–6.218)	44.779 (95%, CI: 31.962–57.595)
KNN	5.064 (95%, CI: 4.156–5.972)	35.924 (95%, CI: 29.750–42.099)
Neural Network	5.067 (95%, CI: 4.741–5.394)	42.686 (95%, CI: 36.702–48.669)

CI: confidence interval; KNN: k-Nearest Neighbors; MAE: mean absolute error; MSE: mean squared error.

**Table 5:** Most important features for each model by LIME.

Model	Features	Value
Random Forest	Tumor_side_right	0.089
	Tumor_side_center	0.045
	volume_ratio_Edema_Total	0.035
XGBoost	Tumor_side_center	0.163
	volume_ratio_AI_model_Manual_normalised	0.091
	volume_ratio_Edema_Necrosis	0.085
KNN	AI_model_Edema	0.271
	Manual_computed_volume	0.070
	AI_model_Contrast	0.034
Neural Network	AI_model_estimated_volume	0.160
	Manual_computed_volume	0.143
	Age_at_diagnosis	0.090

AI: artificial intelligence; KNN: k-Nearest Neighbors; LIME: local interpretable model-agnostic explanations.

**Table 6:** Studies investigating the link between perilesional edema and the prognosis in GB patients.

Study	Purpose	Number of Patients	MRI Sequences	Results
Qin et al., 2021 [59]	Analysis of the impact of PTBE on GB patients	255	T1WI, T2WI, FLAIR	Surgical resection of PTBE tissue was found to reduce midline shift caused by edema. Interestingly, patients who underwent PTBE tissue resection experienced a delay in glioblastoma recurrence compared to those without resection.
Liang et al., 2021 [60]	Debate on the importance of PTBE extent in GB prognosis after high-dose proton boost following tumor resection	45	T2WI, CE-T1WI, FLAIR	Patients with limited PTBE had significantly longer OS and PFS compared to those without limited PTBE.
Wu et al., 2015 [61]	Analysis of the impact on survival in malignant glioma cases	109	T1WI, T2WI, CE-T1WI	Univariate analysis revealed that patients with major PTBE had a significantly shorter survival time compared to patients with minor PTBE. Multivariate analysis confirmed that the extent of PTBE shown by pre-operative MRI was an independent prognostic factor.
Schoenegger et al., 2009 [62]	Evaluation of the prognostic impact of pre-treatment PTBE detected on MRI scans in patients with GB	110	T1WI, T2WI, CE-T1WI, FLAIR	The study found that PTBE on preoperative MRI is an independent prognostic factor, contributing to a more subgroup-oriented treatment approach. Major edema was associated with significantly shorter survival compared to minor edema.

CE-T1WI: contrast-enhanced T1-weightedimage, FLAIR: fluid attenuated inversion recovery, GB: glioblastoma, MRI: magnetic resonance imaging, OS: overall survival, PFS: progression-free survival, PTBE: peritumoral brain edema, T1WI: T1 weighted image, T2WI: T2 weighted image.

## Data Availability

The original contributions presented in this study are included in the article. Further inquiries can be directed to the corresponding author.

## Abbreviations

The following abbreviations are used in this manuscript:

AI	Artificial Intelligence
CE-T1W	T1 Weighted Contrast-Enhanced
CNS	Central Nervous System
DICOM	Digital Imaging and Communication in Medicine
FD	Fractal Dimension
GB	Glioblastoma
LTS	Long-Term Survivors
ML	Machine Learning
MAE	Mean Absolute Error
Max	Maximum Value
Min	Minimum Value
MRI	Magnetic Resonance Imaging
MSE	Mean Squared Error
OS	Overall Survival
PFS	Progression-Free Survival
PTBE	Peritumoral Brain Edema
T2W FLAIR	T2-Weighted Fluid-Attenuated Inversion Recovery
SD	Standard Deviation
T1WI	T1 Weighted Image
T2WI	T2 Weighted Image
WHO	World Health Organization
